# PDK1 plays a vital role on hematopoietic stem cell function

**DOI:** 10.1038/s41598-017-05213-3

**Published:** 2017-07-10

**Authors:** Tianyuan Hu, Cong Li, Le Wang, Yingchi Zhang, Luyun Peng, Hui Cheng, Yajing Chu, Weili Wang, Hideo Ema, Yingdai Gao, Zhenyu Ju, Zhongzhou Yang, Xiaomin Wang, Tao Cheng, Weiping Yuan

**Affiliations:** 1 State Key Laboratory of Experimental Hematology, Institute of Hematology and Blood Diseases Hospital, and Center for Stem Cell Medicine, Chinese Academy of Medical Sciences and Peking Union Medical College, Tianjin, China; 2 Institute of Ageing, Hangzhou Normal University, Hangzhou, China; 3 Ministry of Education Key Laboratory of Model Animal for Disease Study, Model Animal Research Center, Nanjing Biomedical Research Institute, Nanjing University, Nanjing, China; 4 Department of Molecular and Human Genetics, Baylor College of Medicine, Houston, TX 77030 USA; 5 Department of Pediatrics, University of Texas Health Science Center at Houston, Houston, TX 77030 USA

## Abstract

3-Phosphoinositide-dependent protein kinase 1 (PDK1) is a pivotal regulator in the phosphoinositide 3-kinase (PI3K)-Akt signaling pathway that have been shown to play key roles in the functional development of B and T cells via activation of AGC protein kinases during hematopoiesis. However, the role of PDK1 in HSCs has not been fully defined. Here we specifically deleted the *PDK1* gene in the hematopoietic system and found that *PDK1*-deficient HSCs exhibited impaired function and defective lineage commitment abilities. Lack of *PDK1* caused HSCs to be less quiescent and to produce a higher number of phenotypic HSCs and fewer progenitors. *PDK1*-deficient HSCs were also unable to reconstitute the hematopoietic system. Notably, HSC function was more dependent on PDK1 than on mTORC2, which indicates that PDK1 plays a dominant role in the Akt-mediated regulation of HSC function. *PDK1*-deficient HSCs also exhibited reduced ROS levels, and treatment of *PDK1*-deficient HSCs with L-butathioninesulfoximine *in vitro* elevated the low ROS level and promoted colony formation. Therefore, PDK1 appears to contribute to HSC function partially via regulating ROS levels.

## Introduction

Hematopoietic stem cells (HSCs) exist as a rare self-renewing population that gives rise to hematopoietic progenitor and mature cells. HSCs are tightly regulated to maintain the balance between self-renewal, proliferation and differentiation in response to environmental cues. The elucidation of the mechanisms of HSC function is valuable to fully understand the hematopoietic process and HSC-related clinical applications.

The PI3K-Akt signaling pathway plays essential roles in the regulation of hematopoiesis^1^. Extracellular signals activate PI3K, which generates the second messenger phosphatidylinositol 3,4,5-trisphosphate (PtdIns(3,4,5)P_3_) for subsequent action. The downstream Akt is then recruited to the plasma membrane and activated by phosphorylation at its S473 and T308 residues by mTORC2 and 3-phosphoinositide-dependent protein kinase 1 (PDK1), respectively^2, 3^. Activated Akt regulates multiple biological processes, including cell survival, proliferation and protein synthesis via downstream effectors^4^. Both mTORC2 and PDK1 are likely required for full Akt activation^5^. Previous study found that down-regulated PI3K activity impaired the reconstitution of HSCs^6^. Furthermore, deletion of PTEN in hematopoietic cells depleted HSC pool by promoting its differentiation and proliferation^7^. The downstream molecules also involved in the regulation of HSC function. For example, FoxO family proteins control HSC quiescence by regulating ROS levels^8^. Akt, a central factor in this pathway, maintains HSC function also by modulating ROS levels^9^.

PDK1 is critical for cell survival and development in many species, including yeast, *C. elegans* ^10^ and *Drosophila* ^11^. PDK1 is also essential for murine embryonic development. Mice lacking the *PDK1* gene die at embryonic day 9.5 and exhibit abnormalities in various tissues^12^. *PDK1* hypomorphic mice exhibit smaller bodies and organ volumes, and conditional deletion of *PDK1* in muscle cells results in cardiac defects and a shortened lifespan^13^. T cell stage-specific deletion of *PDK1* causes a T cell differentiation blockade and a significant decrease in T cell numbers in the thymus at the DN4 stage^14^. PDK1 is also required for B cell development and survival since the ablation of *PDK1* in the hematopoietic system causes stalled B cell development and impaired B cell VDJ recombination^15, 16^. These findings suggest that PDK1 defines the functions and development of hematopoietic cells, including T cells and B cells.

However, the specific role(s) of PDK1 in the regulation of HSCs has not been fully delineated. In this study, we conditionally deleted *PDK1* in a murine hematopoietic system and found that *PDK1* deletion impaired the reconstitution capacity of HSCs and led to an impaired hematopoiesis. We also demonstrated that PDK1 regulated HSC function probably through controlling cellular ROS levels.

## Materials and Methods

All experiments were carried out in accordance with the guidelines approved by the Institute of Hematology and Blood Diseases Hospital, Chinese Academy of Medical Science.

### Mice

*PDK1* ^*fl/fl*^ and *Rictor* ^*fl/fl*^ mice were generously provided by Drs. Dario R. Alessi^12^ and Mark A. Magnuson^17^, respectively. All mice were backcrossed for ten generations onto a C57BL/6 (CD45.2^+^) background. *PDK1* ^*fl/fl*^ and/or *Rictor* ^*fl/fl*^ mice were crossed with Vav-Cre mice to delete *PDK1* or *Rictor* in hematopoietic cells. The Institutional Animal Care and Use Committee (IACUC) of the Institute of Hematology and Blood Diseases Hospital, Chinese Academy of Medical Science approved all animal procedures, and the mice were housed in the SPF facilities in the same institute.

### Flow cytometry analysis

Single-cell suspensions from blood, spleen or bone marrow were isolated, washed and stained using fluorochrome-labeled antibodies (BD Biosciences) based on the expression of surface or intracellular markers. All flow cytometry experiments were performed using either FACS Canto II or LSR II (BD Biosciences), and the data were analyzed using the FlowJo software.

### Cell separation using MACS and FACS

Lineage-positive cells were pre-depleted from bone marrow cells using the MACS system (Miltenyi Biotec, Sunnyvale, CA, USA) for LT-HSC, ST-HSC and MPP cell isolation. The remaining cells were sequentially stained for LT-HSC, ST-HSC and MPP markers. The cells were sorted after staining using a FACS Aria III cytometer (BD Biosciences).

### Bone marrow transplantation

For bone marrow transplantations, 1 × 10^6^ freshly isolated C57BL/6 (CD45.2^+^) WT, *Vav*-*Cre*;*PDK1* ^*fl/fl*^ (PDK1^Δ/Δ^), *Vav-Cre*;*Rictor* ^*fl/fl*^ (Rictor^Δ/Δ^) and DKO (Rictor^Δ/Δ^PDK1^Δ/Δ^) cells were suspended in PBS and injected into the tail veins of lethally irradiated BL.SJL (CD45.1^+^) recipient mice (950 rad in 2 doses, 4 h apart). For competitive bone marrow transplantation experiments, 0.5 × 10^6^ freshly isolated cells from WT, Rictor^Δ/Δ^, PDK1^Δ/Δ^ or Rictor^Δ/Δ^PDK1^Δ/Δ^ mice (CD45.2^+^) and 0.5 × 10^6^ competitive cells (CD45.1^+^) were suspended in PBS and injected into the tail veins of lethally irradiated CD45.1^+^ recipient mice. Peripheral blood cells were collected 4, 8, 12, 16, 20 and 24 weeks after transplantation, and bone marrow cells were collected 16 and 24 weeks after transplantation for further analyses. Bone marrow transplantation and competitive bone marrow transplantation experiments were performed using 3 mice for each time point. For HSC transplantation experiments, 300 sorted HSCs from WT and PDK1^Δ/Δ^ mice (CD45.2^+^) and 2 × 10^5^ competitive cells (CD45.1^+^) were injected into the tail veins of lethally irradiated CD45.1^+^ recipient mice. Bone marrow cells were collected 1.5 and 3 months after transplantation for further analyses.

### Cell cycle analysis

Freshly isolated BM cells were stained using antibodies against Sca-1, c-kit, CD34, Flt3 and lineage markers to identify HSCs and MPPs. Antibody-labeled cells were subsequently incubated with DAPI and Ki67 to determine the cell cycle profile. The Ki67 antibody allows for the separation of cells in G0 and G1 stages, and co-staining with DAPI allows for the separation of S/G2/M cell populations. Cells were analyzed using a LSR II flow cytometer (BD Biosciences).

### Brdu staining assay

1 × 10^6^ LSK (Lin^−^c-kit^+^Sca-1^+^) were cultured in SFEM (Gibco) for 12 h, washed three times in 0.1 M phosphate buffered saline (PBS), and incubated with BrdU for 2 h. BrdU labeling assays were performed using the FITC-BrdU Flow kit (BD Biosciences) according to the manufacturer’s instruction.

### Apoptosis assay

BM cells from groups were incubated with antibodies against Sca-1, c-kit, CD34, Flt3 and linage markers to identify HSCs and MPPs. Antibody-labeled cells were washed and incubated with Annexin V and DAPI at room temperature followed by flow cytometry analysis using an LSR II flow cytometer.

### Measurement of ROS

BM cells were incubated with antibodies against Sca-1, c-kit, CD34, Flt3 and linage markers to identify HSCs and MPPs. Antibody-labeled cells were washed and incubated in 10 µM DCF-DA for 20 minutes at 37 °C for flow cytometric analysis^18, 19^. BSO (L-butathioninesulfoximine, Sigma-Aldrich) was added to MethoCult GF M3434 medium at various concentrations (0.01 μM, 0.02 μM, 0.03 μM, 0.05 μM, 0.1 μM or 0.2 μM) to increase ROS levels *in vitro* for further CFC analysis^9^.

### Real-time RT-PCR

mRNA expression levels were quantified using real-time RT-PCR with SYBR Green PCR Master Mix. Changes in relative gene expression between groups were calculated using the 2^−∆∆CT^ method normalized to *GAPDH* expression.

### Colony-forming cell (CFC) assay

A total of 2 × 10^4^ bone marrow cells from WT and *Vav-Cre;PDK1* ^*fl/fl*^ mice were plated in MethoCult GF M3434 (Stem Cell Technologies) medium containing various cytokines to support the hematopoietic progenitors. For HSC and MPP colony-forming assays, 300 HSCs from WT and *Vav-Cre;PDK1* ^*fl/fl*^ mice were plated in MethoCult GF M3434 (Stem Cell Technologies) medium. Colonies were counted after 3–14 days of culture according to the manufacturer’s instructions.

### Statistical analyses

Significant differences in parameters were assessed between groups using unpaired Student’s test. Significance is denoted with asterisks (*P < 0.05, **P < 0.01, ***P < 0.001), and P > 0.05 was considered non-significant (NS).

## Results

### *PDK1* deficiency in mice results in increased phenotypic HSCs and decreased progenitor cells

**Figure 1:**
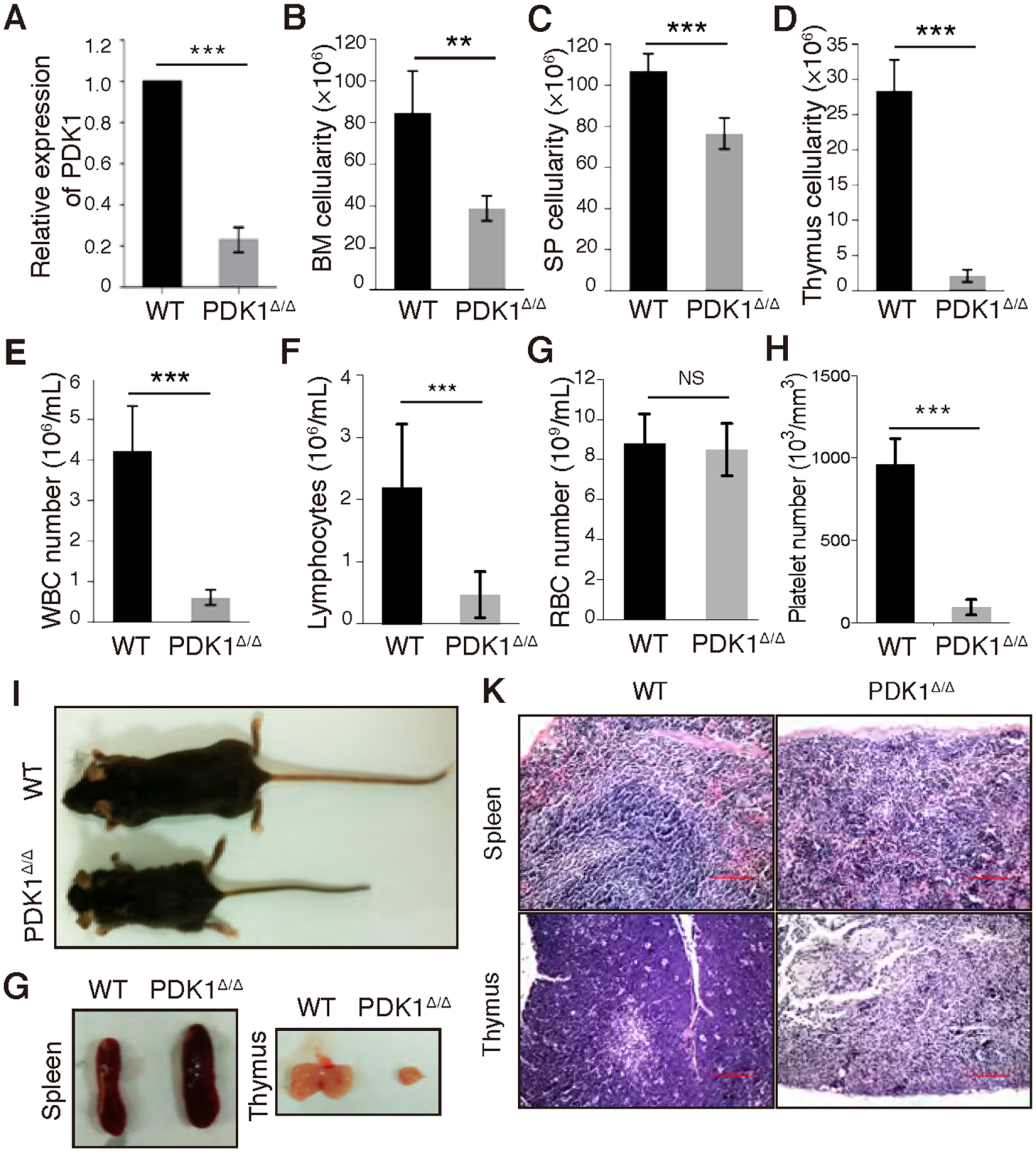
Phenotype analysis of *PDK1-*deficient mice. (**A**) Low expression of PDK1 mRNA in PDK1^Δ/Δ^ BM cells as examined using real-time PCR. (**B**–**D**) The BM, spleen and thymus cell counts in WT and PDK1^Δ/Δ^ mice. (**E**) White blood cell (WBC) counts in PB from 3–4 week WT and PDK1^Δ/Δ^ mice. (**F**–**H**) Lymphocyte, Red blood cell (RBC), and platelet counts in PB from 3–4-week WT and PDK1^Δ/Δ^ mice. (**I**–**G**) Representative images of the mouse body, spleen and thymus of WT and PDK1^Δ/Δ^ mice. (**K**) H&E staining of the spleen and thymus from WT and PDK1^Δ/Δ^ mice.

We generated *PDK1* conditional knockout mice *Vav-Cre*;*PDK1* ^*fl/fl*^ (PDK1^Δ/Δ^) to explore the roles of PDK1 in murine HSCs. *PDK1* ^*fl/fl*^ (WT) mice were used as a control. Real-time PCR confirmed the efficient excision of the *PDK1* gene in PDK1^Δ/Δ^ mice (Fig. 1A). The BM cellularity, splenocytes and thymocytes were decreased after *PDK1* deletion (Fig. 1B–D). WBC, lymphocyte and platelet number were also decreased in PDK1 deficient mice (Fig. 1E–H). PDK1^Δ/Δ^ mice were smaller than wild-type controls and exhibited a larger spleen and smaller thymus (Fig. 1G–I). H&E staining revealed evidence of extramedullar hematopoiesis in *PDK1*-deficient spleens (Fig. 1K).

**Figure 2:**
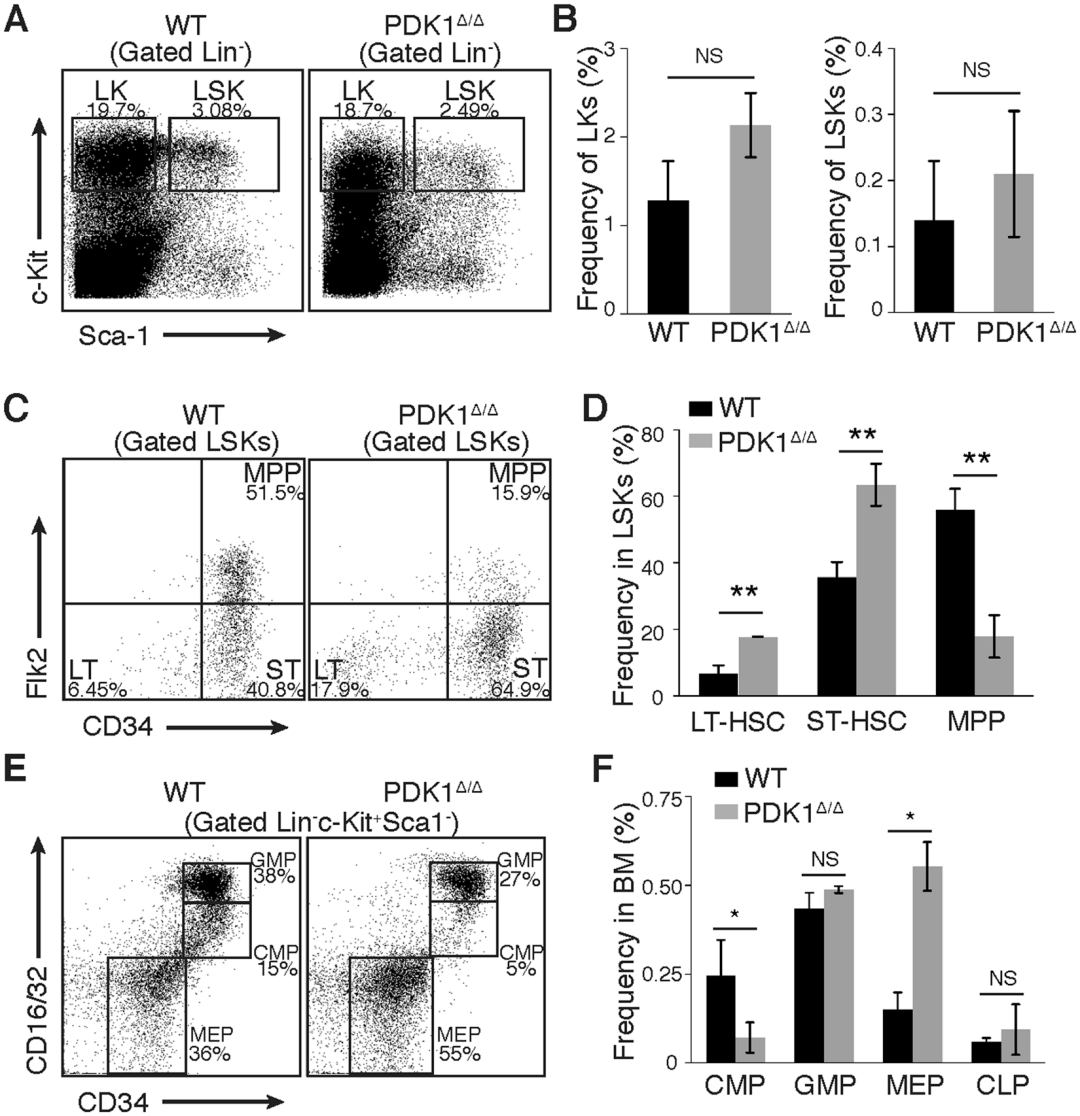
Conditional deletion of *PDK1* in a hematopoietic system results in increased HSCs but reduced progenitors. (**A**,**B**) Representative FACS plots and histograms showing the frequency of LKs (Lin^−^c-kit^+^Sca-1^−^ cells) and LSKs (Lin^−^c-kit^+^Sca-1^+^ cells) in BM from WT and PDK1^Δ/Δ^ mice. (**C**,**D**) Representative FACS plots and histograms showing the frequency of LT-HSCs, ST-HSCs and MPPs in LSKs from WT and PDK1^Δ/Δ^ mice. (**E**) Representative FACS plots showing the frequency of GMPs, CMPs and MEPs in BM from WT and PDK1^Δ/Δ^ mice. (**F**) Representative histograms showing the frequency of CMPs, GMPs, MEPs and CLPs in LKs from WT and PDK1Δ/Δ mice. The data are shown as the mean ± SD (n = 5); *P < 0.05; **P < 0.01; ***P < 0.001; NS, not significant.

FACS analysis revealed that the percentage of LSK (Lin^−^c-kit^+^Sca-1^+^) cells and LK (Lin^−^c-kit^+^Sca-1^−^) cells in PDK1^Δ/Δ^ mice were comparable to those of the control mice (Fig. 2A,B). Further examination of the frequency of HSCs and HPCs in BM using flow cytometry revealed significant increases in phenotypic LT-HSCs and ST-HSCs but substantial decreases in MPPs (Fig. 2C,D, Figure S1A,B) and CMPs after *PDK1* deletion (Fig. 2E,F). These results indicated that the loss of *PDK1* significantly perturbed steady-state hematopoiesis.

### PDK1-deficient HSCs fail to reconstitute the hematopoietic system upon transplantation

**Figure 3:**
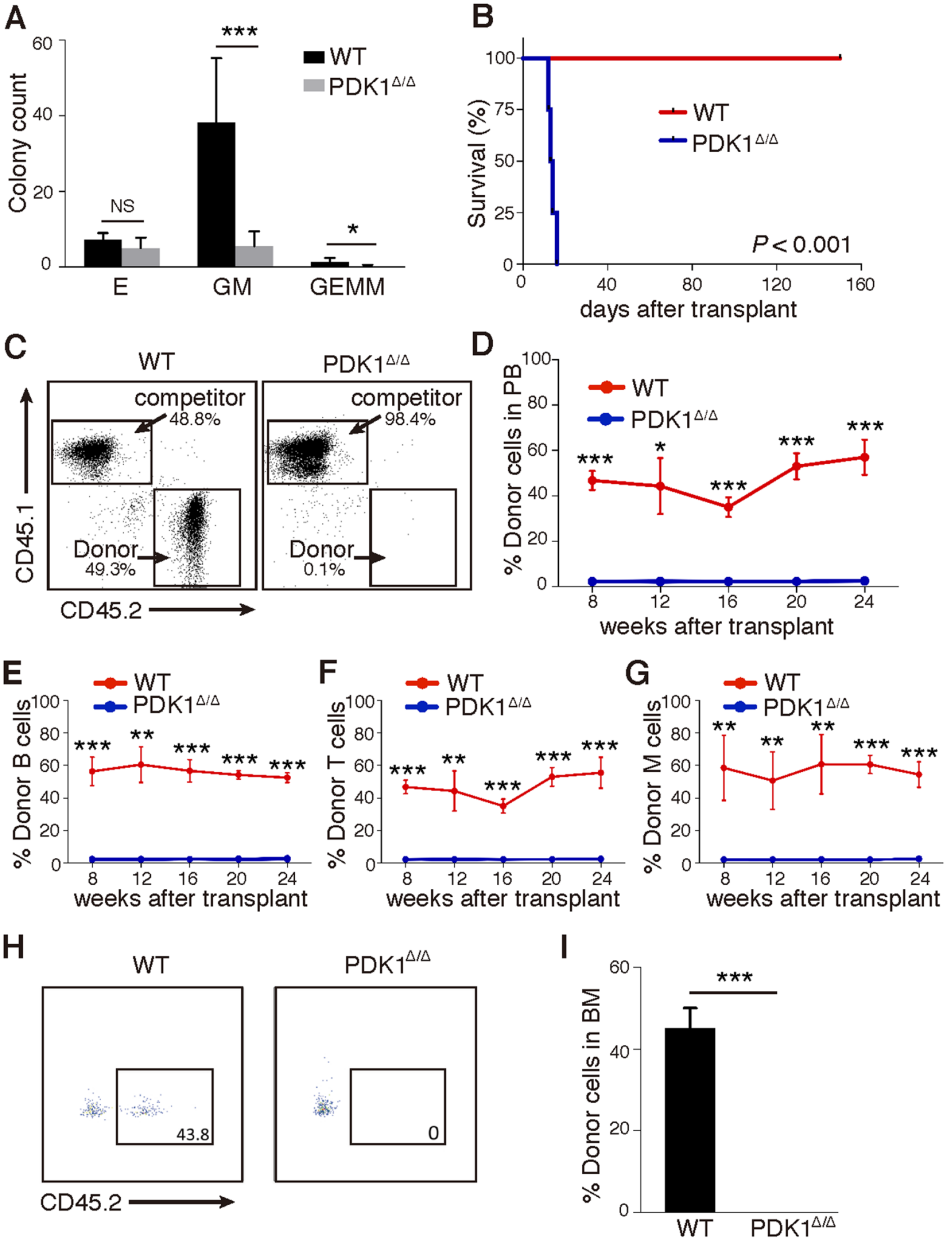
*PDK1* is required for HSC reconstitution upon transplantation. (**A**) Quantification of colony numbers generated by WT and PDK1^Δ/Δ^ BM cells (2 × 10^4^/well) in colony-forming assays. (**B**) Survival curve of lethally irradiated recipient mice (CD45.1^+^) transplanted with whole bone marrow cells from WT and PDK1^Δ/Δ^ mice (CD45.2^+^) (n ≥ 10). (**C**) Whole bone marrow cells from WT and PDK1^Δ/Δ^ mice (CD45.2^+^) were mixed with WT whole bone marrow competitor cells (CD45.1^+^) in a 1:1 ratio and transplanted into lethally irradiated recipients (CD45.1^+^). Representative FACS plots showing the reconstitution proportion of donor cells against competitors in PB 4 months after transplantation. (**D**) PB chimera rates in recipients 8, 12, 16, 20, and 24 weeks after competitive transplantation. (**E**–**G**) Multilineage distribution (B cells, T cells and myeloid cells) of donor cells in PB at the indicated time points. (**H**,**I**) Representative FACS plots showing the reconstitution of donor HSCs against competitors 3 months after transplantation. All data are shown as the mean ± SD (n ≥ 3); *P < 0.05; **P < 0.01; ***P < 0.001; NS, not significant.

Colony-Forming Cell (CFC) assays were performed to determine the colony-forming abilities of *PDK1*-deficient progenitor cells *in vitro* to investigate whether the loss of PDK1 affected their function. PDK1^Δ/Δ^ BM cells gave rise to fewer CFU-GM and CFU-GEMM colonies when compared with control BM cells in MethoCult GF M3434 medium (Fig. 3A), demonstrating that the loss of *PDK1* impairs the colony-forming ability of *PDK1*-deficient cells *in vitro*.

BM cells from PDK1^∆/∆^ and WT mice (CD45.2^+^) were transplanted into lethally irradiated recipients (CD45.1^+^) to evaluate the effect of PDK1 on HSC reconstitution ability (Figure S2A). BM cells from *PDK1*-deficient mice failed to reconstitute the hematopoietic system in recipient mice, while the WT BM cells fully rescued the lethally irradiated mice (Fig. 3B). We didn’t found any significant different in homing assay (Figure S2B), suggesting that the impaired reconstitute ability in recipients by PDK1 knockout BM cells might not be due to their homing defect.

We performed competitive transplantation experiments to further determine the function of PDK1 in HSCs. BM cells from PDK1^∆/∆^ and WT mice (CD45.2^+^) were transplanted into lethally irradiated recipients (CD45.1^+^) with wild-type competitive cells (CD45.1^+^) (Figure S2C). The recipient mice displayed extremely reduced percentages of PDK1^∆/∆^-derived total donor cells, CD3^+^, B220^+^ and myeloid cells in the peripheral blood (PB) at various time points after transplantation (Fig. 3C–G). The chimerism of BM cells was examined using flow cytometric analyses 6 months after transplantation. PDK1^∆/∆^-derived BM cells were almost absent in recipient BM (Figure S2D–F), but the control and competitive cells generated normal proportions of hematopoietic cells. These results suggest that *PDK1*-deficient HSCs fail to reconstitute hematopoiesis *in vivo* upon transplantation.

To determine the HSC function after PDK1 deletion, we transplanted 300 LT-HSCs from PDK1^∆/∆^ or WT mice and competitor cells into lethally irradiated recipient mice. We found that PDK1 deficient HSCs loss the ability to reconstitution in recipients where control group showed the normal self-renew ability (Fig. 3H,I). These results indicate that PDK1 is vital for HSC reconstitution.

### PDK1 deficiency is dominant over mTORC2 deficiency

PDK1 phosphorylates Akt at its T308 residue. Therefore, we examined the related protein phosphorylation levels in LSKs and HSCs using flow cytometry. Phosphorylation at the T308 residue of Akt was lower in PDK1-deficient HSCs (Figure S3A), but the phosphorylation level of S473 was comparable to control (Figure S3B). Notably, a downstream effector of Akt, S6 protein, exhibited decreased phosphorylation levels, which indicates an impairment of Akt signaling transduction after *PDK1* gene loss (Figure S3C). Phosphorylation of P44/P42 and Stat3 was altered after the loss of PDK1, which suggests a potential role of PDK1 in the p38-MAPK and Jak-Stat signaling pathways (Figure S3D,E).

**Figure 4:**
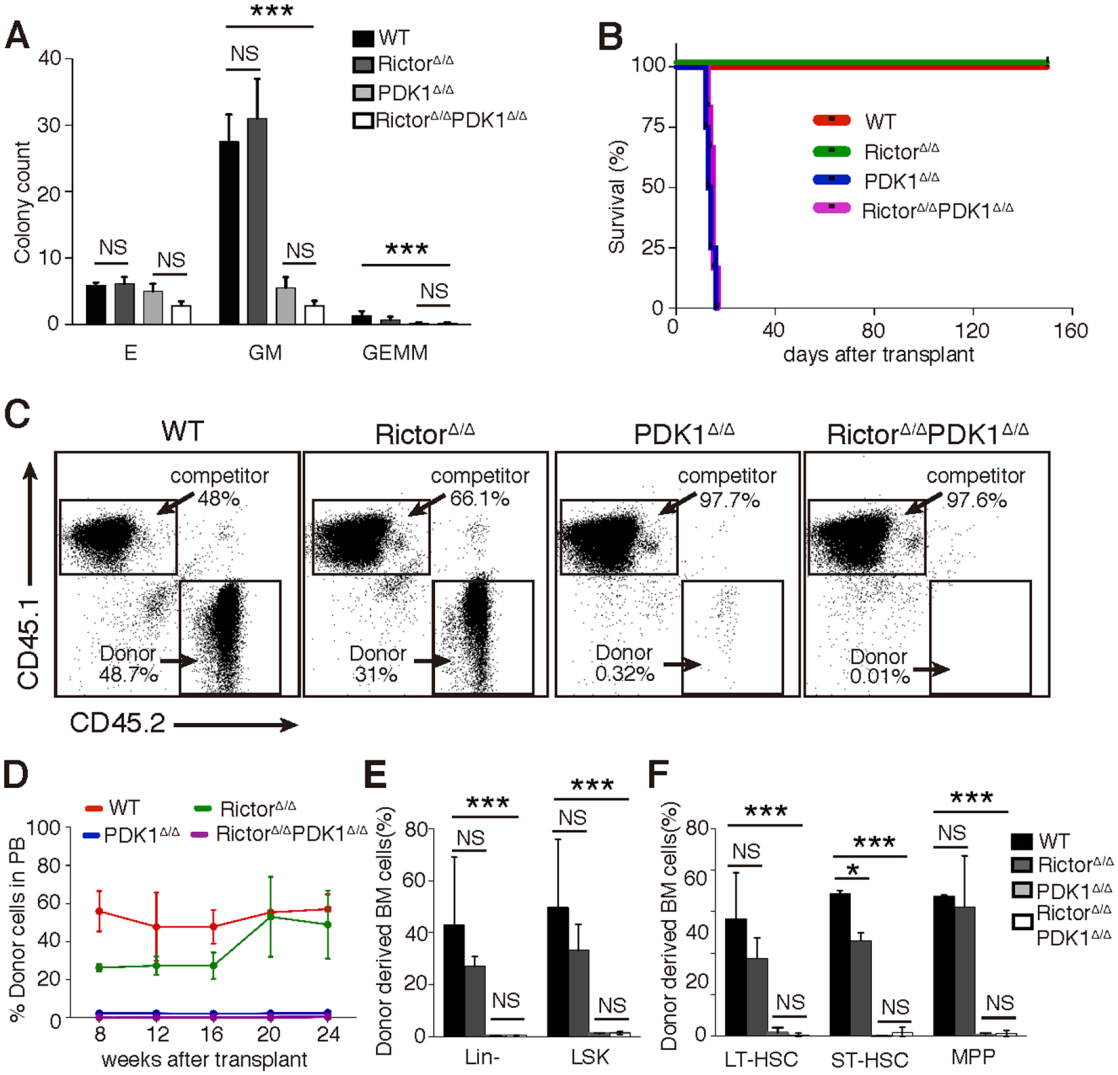
PDK1, but not Rictor, plays a dominant role in Akt-mediated HSC functions. (**A**) Quantification of colony numbers generated by WT, Rictor^Δ/Δ^, PDK1^Δ/Δ^ and Rictor^Δ/Δ^ PDK1^Δ/Δ^ BM cells (2 × 10^4^/well) in colony-forming assays. (**B**) Survival rate of lethally irradiated recipients (CD45.1^+^) transplanted with whole bone marrow cells from WT, Rictor^Δ/Δ^, PDK1^Δ/Δ^ and Rictor^Δ/Δ^PDK1^Δ/Δ^ mice (CD45.2^+^), (n ≥ 10). (**C**) Whole bone marrow cells from WT, Rictor^Δ/Δ^, PDK1^Δ/Δ^ and Rictor^Δ/Δ^PDK1^Δ/Δ^ mice (CD45.2^+^) that were mixed with WT whole bone marrow competitor cells (CD45.1^+^) in a 1:1 ratio were transplanted into lethally irradiated recipients (CD45.1^+^). Representative FACS plots showing the reconstitution portion of donor cells against competitors in PB. (**D**) PB chimera rates in recipients 8, 12, 16, 20, and 24 weeks after competitive transplantation. (**E,F**) The frequencies of donor-derived BM Lin^−^ cells, LSKs, LT-HSCs, ST-HSCs and MPPs in recipients. The data are shown as the mean ± SD (n ≥ 3); *P < 0.05; ***P < 0.001; NS, not significant.

To explore how mTORC2 and/or PDK1 influence Akt function in HSCs, we generated Rictor^Δ/Δ^PDK1^Δ/Δ^ (DKO) mice in conjunction with Rictor^Δ/Δ^ and PDK1^Δ/Δ^ mice to explore how mTORC2 and/or PDK1 influence Akt function in HSCs. In addition to the defective colony-forming ability of Rictor^Δ/Δ^PDK1^Δ/Δ^ progenitors (Fig. 4A), lethally irradiated recipient mice transplanted with whole bone marrow cells from PDK1^Δ/Δ^ or Rictor^Δ/Δ^PDK1^Δ/Δ^ mice failed to survive compared with WT or Rictor^Δ/Δ^ BM cell transplantations (Fig. 4B). Competitive transplantation experiments revealed an impaired reconstitution ability of Rictor^Δ/Δ^PDK1^Δ/Δ^ HSCs after transplantation (Fig. 4C–F), which indicates a long-term hematopoiesis defect after *Rictor/PDK1* deletion, consistent with PDK1^Δ/Δ^ HSCs. Therefore, our data suggest that PDK1 plays a dominant role in the Akt-mediated regulation of HSC function compared with Rictor/mTORC2.

### PDK1 deficiency results in less quiescent HSC

**Figure 5:**
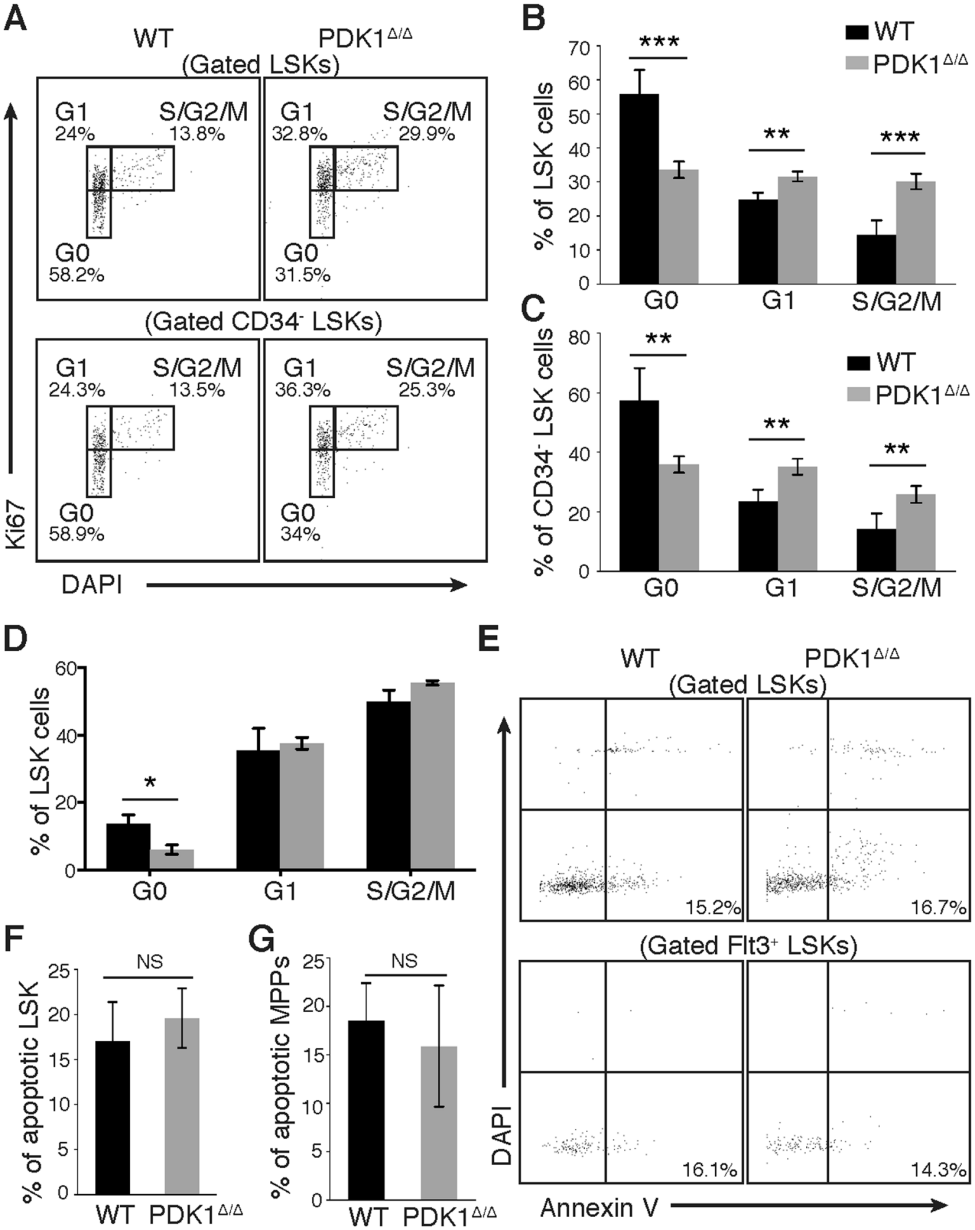
*PDK1* deficiency leads to a loss of HSC quiescence. (**A**) Representative FACS plots showing DAPI and Ki67 staining profiles in LSK cells (upper panel) and CD34^−^LSK cells (lower panel). (**B**,**C**) Histograms showing the cell cycle status of LSKs and CD34^−^LSKs. (**D**) Histograms showing the Brdu incorporation status of LSKs. (**E**) Representative FACS plots showing DAPI and Annexin V staining profiles of LSK cells (upper panel) and Flt3^+^LSK cells (lower panel). (**F**,**G**) Histograms showing cell apoptosis status of LSKs and Flt3^+^LSKs. The data are shown as the mean ± SD (n = 5); **P < 0.01; ***P < 0.001; NS, not significant.

We examined the cell cycle status of HSCs using Ki67 to categorize HSCs in resting or active cell cycle stages during cellular proliferation to explore the mechanism of PDK1 regulation of HSCs. The percentage of cells in G0 stage was decreased significantly in PDK1^∆/∆^ HSCs compared with control HSCs, and this result was characterized by a reduction in the Ki67^−^ G0 fraction (Fig. 5A–C). PDK1^∆/∆^ HSCs were also enriched in G1 and S/G2/M phases, which suggest an increase in HSC exit from their quiescent state (Fig. 5C). We further confirmed this in an *in vitro* Brdu incorporation assay and found that PDK1 deficiency decreased the G0 fraction of LSKs (Fig. 5D). Next, we examined the proportion of HSC undergoing apoptosis. We found a comparable percentage of Annexin V^+^DAPI^−^ PDK1^∆/∆^ and WT HSCs (Fig. 5E–G, Figure S4A,B). These findings indicate that the loss of PDK1 altered HSC cell cycle status to be less quiescent.

### PDK1-deficient HSCs show reduced colony-forming ability and decreased ROS levels

**Figure 6:**
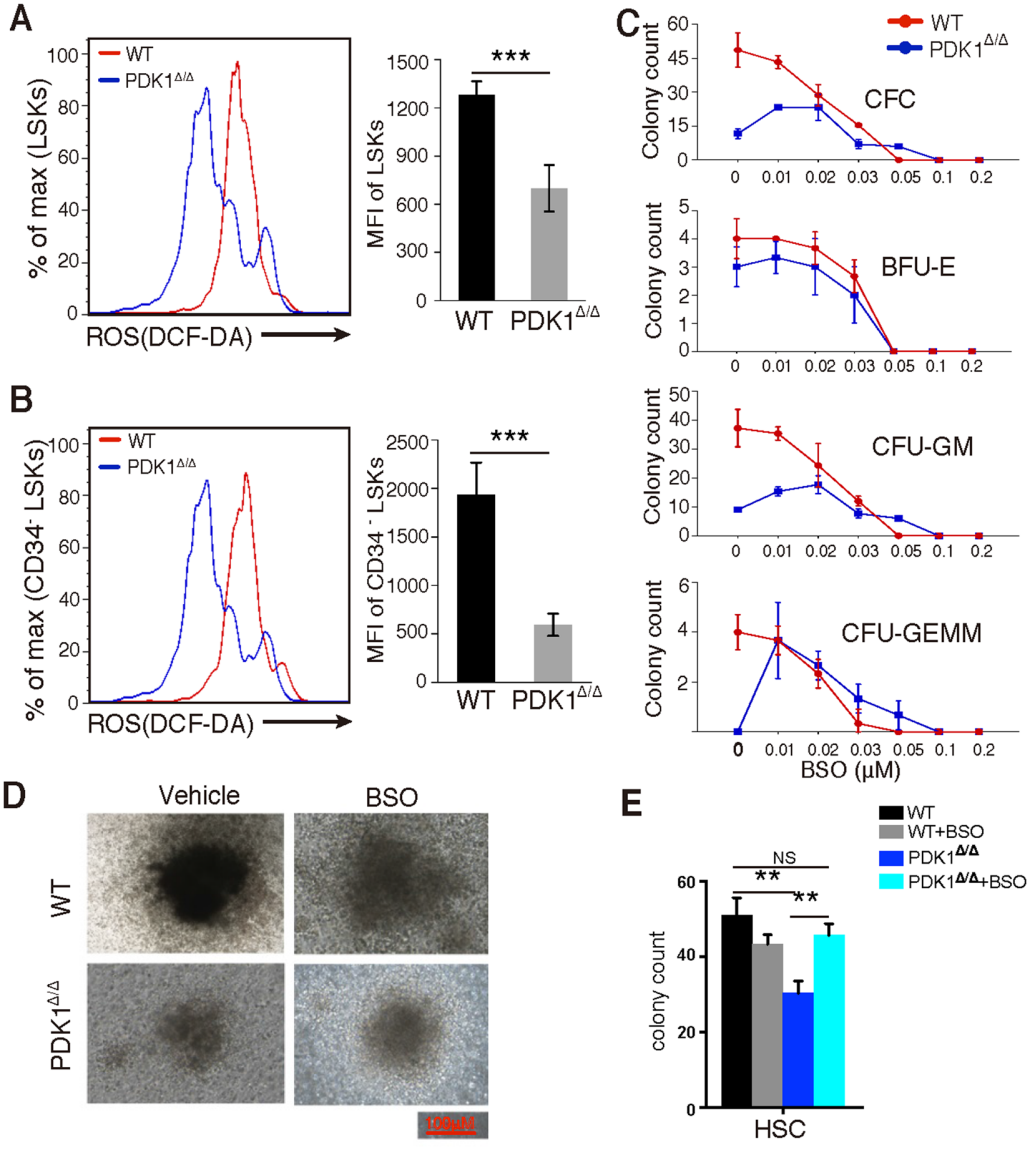
*PDK1*-deficient HSCs display lower ROS levels than controls. (**A**,**B**) LSKs (**A**) and CD34^−^LSKs (**B**) from WT or PDK1^Δ/Δ^ mice were stained with DCF-DA and analyzed using flow cytometry. Representative histograms showed the MFI (median fluorescence intensities) of DCF-DA-labeled cells from WT or PDK1^Δ/Δ^ mice. (**C**) BM cells from WT or PDK1^Δ/Δ^ mice were treated with BSO at various concentrations for colony-forming assays. (**D**,**E**) 300 HSCs were sorted from WT or PDK1^Δ/Δ^ mice and treated with BSO (0.02 μM) for colony-forming assays. The data are shown as the mean ± SD (n = 5); *P < 0.05; **P < 0.01; ***P < 0.001; NS, not significant.

Previous studies demonstrated that ROS levels correlate with HSC quiescence^8, 20, 21^. Therefore, we assessed ROS levels in *PDK1*-deficient HSCs by measuring intracellular ROS levels using 2′-7′-dichlorofluorescein diacetate (DCF-DA) staining^22, 23^. Notably, we found that *PDK1*-deficient LSKs and HSCs exhibit significantly reduced ROS levels compared to that of control HSCs (Fig. 6A,B).

We treated *PDK1*-deficient BM cells with various concentrations of BSO *in vitro* to increase cellular ROS levels and examined the colony-forming ability of HSCs after treatment to probe whether reduced cellular ROS levels were responsible for the impaired function of PDK1^Δ/Δ^ hematopoietic stem and progenitor cells. The colony counts of *PDK1*-deficient BM cells treated with 0.01 μM and 0.02 μM BSO increased significantly, which indicates recovery of the colony-forming ability with increasing ROS levels (Fig. 6C). Notably, the recovery effect was only observed with BSO concentrations lower than 0.03 μM. Next we raised ROS level by BSO treatment with sorted HSCs *in vitro*. We found the increased colony size (Fig. 6D) and number (Fig. 6E) of PDK1-deleted HSCs upon BSO treatment, indicating the impaired colony-forming ability of PDK1-deficient HSCs was partially rescued by increased ROS level.

## Discussion

We used conditional deletion of *PDK1* gene in a hematopoietic system and found that the loss of PDK1 resulted in an impaired colony-forming ability *in vitro* and a defective short-term and long-term reconstitution ability after transplantation (Fig. 3). HSC quiescence is essential for their self-renewal ability^21, 24, 25^ and the maintenance of HSC functions. Disturbed quiescence of HSCs impairs HSC functions^22, 26^. Therefore, the disrupted cell cycle status, as detected in PDK1-deficient HSCs, may account for the impaired reconstitution ability of HSCs. We observed a reduced G0 phase of HSCs and an increased S/G2/M phase of PDK1^Δ/Δ^ HSCs, which was accompanied by an increase in HSC frequency. These results indicate that PDK1-deficient HSCs were less quiescent due to PDK1 loss. Fewer G0-phase HSCs in PDK1-deficient mice led to reduced HSC reconstitution ability, and the increased HSC proliferation likely occurs through feedback mechanisms because PDK1 deletion resulted in a significant loss of progenitor cells, mature B cells and T cells. This result is consistent with a previous study that HSCs in the G0 phase exhibited enhanced reconstitution ability than less quiescent HSCs^27^.

Lower HSC cellular ROS levels have been demonstrated to be essential for the maintenance of quiescent HSCs^8^. Notably, we found that PDK1-deficient HSCs exhibited lower ROS levels with an increased proportion of HSCs entering the cell cycle. This result likely occurred because the loss of PDK1 either interrupted the regulatory mechanism of adequate HSC ROS level maintenance or perturbed cell cycle regulation independently of ROS, which resulted in the loss of quiescence in HSCs. Moreover, we found that the colony count of PDK1-deficient cells *in vitro* increased when ROS levels were elevated by 0.01–0.02 μM BSO treatment, but the number of colonies decreased when BSO concentrations were above 0.03 μM (Fig. 6). This result suggests that ROS levels are precisely controlled in hematopoietic stem and progenitor cells, and that higher or lower ROS levels beyond the normal range are harmful to hematopoietic stem and progenitor cell functions and PDK1 plays an important role in this process. However, additional work is needed to completely elucidate the roles of ROS in HSCs.

Akt is a major downstream effector of PDK1. A previous study demonstrated that *Akt1/Akt2* double-knockout HSCs exhibited only modest reduced reconstitution ability^9^. Here we showed that *PDK1*-deficient HSCs alone could not reconstitute the recipient mice, whereas Rictor/mTORC2-deficient HSCs successfully reconstituted hematopoiesis in lethally irradiated mice with minor defects in B cell and T cell differentiation^28, 29^. We generated Rictor^Δ/Δ^PDK1^Δ/Δ^ to explore the possible differential downstream signaling roles of PDK1 and mTORC2 on HSCs that govern Akt activation. Notably, *Rictor/PDK1* double-deficient HSCs exhibited very similar phenotypes as *PDK1*-deficient HSCs (Figs 3 and 4). This result indicates that PDK1 plays a dominant role in the Akt-mediated regulation of HSCs. The functional discrepancies in PDK1 and Akt-deficient HSCs may be attributed to other AGC kinases that are regulated by PDK1^5^. Other AGC kinases and PDK1 substrates, such as SGK and p70S6K might also contribute to the defective HSC function after PDK1 deletion^6^. Future studies about other potential downstream factors of PDK1 will improve the current understanding of the role of PDK1 on HSC function.

## Electronic supplementary material

Supplemental Data
