# Supplementary material for: PDK1 plays a vital role on hematopoietic stem cell function
Source: Sci Rep. 2017 Jul 10;7:4943. doi: 10.1038/s41598-017-05213-3 (PMC5504031; doi:10.1038/s41598-017-05213-3)
Supplement: Supplementary file 1 — Supplemental Data [file 41598_2017_5213_MOESM1_ESM.doc]

**Supplemental Data (SREP-16-20646)**

**PDK1 plays a vital role on hematopoietic stem cell function**

Tianyuan Hu^1^**^†‡^**, Cong Li^1^**^†§^**, Le Wang^1^**^†^**, Yingchi Zhang^1^, Luyun Peng^1^, Hui Cheng^1^, Yajing Chu^1^, Weili Wang^1^, Hideo Ema^1^, Yingdai Gao^1^, Zhenyu Ju^2^, Zhongzhou Yang^3^, Xiaomin Wang^1*^, Tao Cheng^1^ and Weiping Yuan^1*^

^1^State Key Laboratory of Experimental Hematology, Institute of Hematology and Blood Diseases Hospital, and Center for Stem Cell Medicine, Chinese Academy of Medical Sciences and Peking Union Medical College, Tianjin, China.

^2^Institute of Ageing, Hangzhou Normal University, Hangzhou, China.

^3^Ministry of Education Key Laboratory of Model Animal for Disease Study, Model Animal Research Center, Nanjing Biomedical Research Institute, Nanjing University, Nanjing, China.

**^†^** These authors contributed equally to this work.

**^‡^** Current address: Department of Molecular and Human Genetics, Baylor College of Medicine, Houston, TX 77030, USA.

**^§^** Current address: Department of Pediatrics, University of Texas Medical School, Houston, TX 77030, USA.

*Correspondence: [wangxiaomin@ihcams.ac.cn](mailto:wangxiaomin@ihcams.ac.cn) or [wpyuan@ihcams.ac.cn](mailto:wpyuan@ihcams.ac.cn)

State Key Laboratory of Experimental Hematology, Institute of Hematology and Blood Diseases Hospital and Center for Stem Cell Medicine, Chinese Academy of Medical Sciences and Peking Union Medical College, Tianjin 300020, China.

Tel/Fax：022-2390-9166/9093, Phone: (86)22-23909418

**Key words:** PDK1, Rictor, Akt, HSC

**Figure S1. Phenotype analysis of *PDK1-*deficient mice.** (A-B) Representative FACS plots and histograms showing the frequency of HSCs (Lin^-^c-kit^+^Sca-1^+^ CD150^+^CD48^-^) from WT and PDK1^Δ/Δ^ mice. The data are shown as the mean±SD (n=5); **P<0.01.

**Figure S2. *PDK1* deficiency impairs long-term hematopoiesis.** (A) For bone marrow transplantation experiments, 1×10^6^ freshly isolated WT or PDK1^Δ/Δ^ cells were injected into the tail veins of lethally irradiated CD45.1^+^ recipient mice. (B) 2×10^6^ lin^-^ cells (CD45.2^+^) were injected into the tail veins of lethally irradiated CD45.1^+^ recipient mice. 16 hour after transplantation, mice were sacrificed and BM cells were isolated to examining the homing ability. (C) For competitive bone marrow transplantation experiments, 0.5×10^6^ freshly isolated WT or PDK1^Δ/Δ^ cells (CD45.2^+^) and 0.5×10^6^ competitive cells (CD45.1^+^) were injected into the tail veins of lethally irradiated CD45.1^+^ recipient mice. (D-F) Bone marrow cells were collected 24 weeks after competitive transplantation for flow cytometric analyses (n≥10); *P<0.05; **P<0.01; ***P<0.001; NS, not significant.

**Figure S3. Protein phosphorylation level analyses for PDK1-related pathways in *PDK1*-deficient HSCs using flow cytometry.** (A-E) Flow cytometry analyses and MFI of pAKT308, pAKT473, pS6, pP44 and pStat3 in LSKs and CD34- LSKs from WT and PDK1^Δ/Δ^ mice. The data are shown as the mean ± SD (n≥3); *P<0.05; **P<0.01; ***P<0.001; NS, not significant.

**Figure S4. Cell apoptosis analysis of PDK1-difficient HSCs.** (A) Representative FACS plots showing DAPI and Annexin V staining profiles in HSC cells. (B) Histograms showing the cell apoptosis status of HSCs. The data are shown as the mean ± SD (n=5); NS, not significant.
